# Doxorubicin Toxicity and Recent Approaches to Alleviating Its Adverse Effects with Focus on Oxidative Stress

**DOI:** 10.3390/molecules30153311

**Published:** 2025-08-07

**Authors:** Lyubomira Radeva, Krassimira Yoncheva

**Affiliations:** Faculty of Pharmacy, Medical University of Sofia, 1000 Sofia, Bulgaria; l.radeva@pharmfac.mu-sofia.bg

**Keywords:** doxorubicin, toxicity, antioxidants, encapsulation, nanoparticles, co-administration

## Abstract

Despite the significant antitumor potential of doxorubicin and its widespread use in the treatment of various oncological diseases, its application is associated with side effects, among which the most common are cardiotoxicity, hepatotoxicity, nephrotoxicity, neurotoxicity, and gonadotoxicity. In contemporary times, innovative strategies to overcome the toxicity of doxorubicin and improve the effectiveness of therapies are intensively researched. The aim of this review is to discuss different approaches to alleviate the common toxic effects of doxorubicin, with an emphasis on oxidative stress. In particular, the review analyzes the significance of pharmaceutical nanotechnology for reducing doxorubicin toxicity while maintaining its antitumor effect (e.g., encapsulation of doxorubicin in passively and/or actively targeted nanoparticles to tumor tissue and cells). Other strategies commented in the review are the simultaneous delivery of doxorubicin with antioxidants and the administration of its derivatives with lower toxicity.

## 1. Introduction

The anthracycline antibiotic doxorubicin is a well-known chemotherapeutic which is widely applied clinically. The mechanisms behind its anticancer effects are related to the inhibition of the enzyme topoisomerase II, which is essential for the survival of cancer cells, characterized with rapid division. The enzyme is responsible for the condensation of chromosomes, decatenation of intertwined DNA strands, and the relaxation of tension in the DNA strand in front of the replication fork. It is known that the anthracyclines form complexes with the enzyme-DNA, leading to DNA damage, cell cycle arrest, and cell death. Moreover, doxorubicin intercalates into the DNA helix leading to the inhibition of DNA and RNA synthesis. The induction of oxidative stress is also part of the effects of doxorubicin. Its quinone moiety can be transformed into a semiquinone by cytochrome P450 reductases, xanthine oxidase, and NADH dehydrogenase (complex I). This leads to the transformation of oxygen into ROS (reactive oxygen species), O^2−^ (superoxide anion), and H_2_O_2_ (hydrogen peroxide). Moreover, the aforementioned ROS could be converted into more reactive hydroxyl radicals (•OH). Increase in the cellular levels of iron could also occur due to interaction with iron regulatory proteins or acceleration of the release of iron from ferritin. This further amplifies iron-mediated oxidative stress [1,2,3]. However, this generation of oxidative stress could lead to the manifestation of toxic effects. Taking this into consideration, the co-administration of antioxidants along with doxorubicin could be considered an appropriate strategy for alleviating its toxicities.

The aim of the present review is to discuss different approaches to alleviate the common toxic effects of doxorubicin, with an emphasis on oxidative stress.

## 2. Search Strategy and Methodology

The Google Scholar search engine, which includes a variety of databases, was used for the search of data for the present review in the year interval 2021–2025. Search terms included “doxorubicin”, “cardiotoxicity”, “neurotoxicity”, “nephrotoxicity”, “hepatotoxicity”, “gonadotoxicity”, “nanoparticles”, “nanoencapsulaion”, “antioxidant”, “double loading”, “derivatives”, and their combinations. Papers that cover drug delivery systems and their relation with in vitro or in vivo toxicity were exclusively selected. Review articles were only used as reference for the subsections regarding toxicity. Other types of papers such as conference proceedings, book chapters and therapeutic strategies were excluded from our sources.

## 3. Toxicity of Doxorubicin

**Figure 1:**
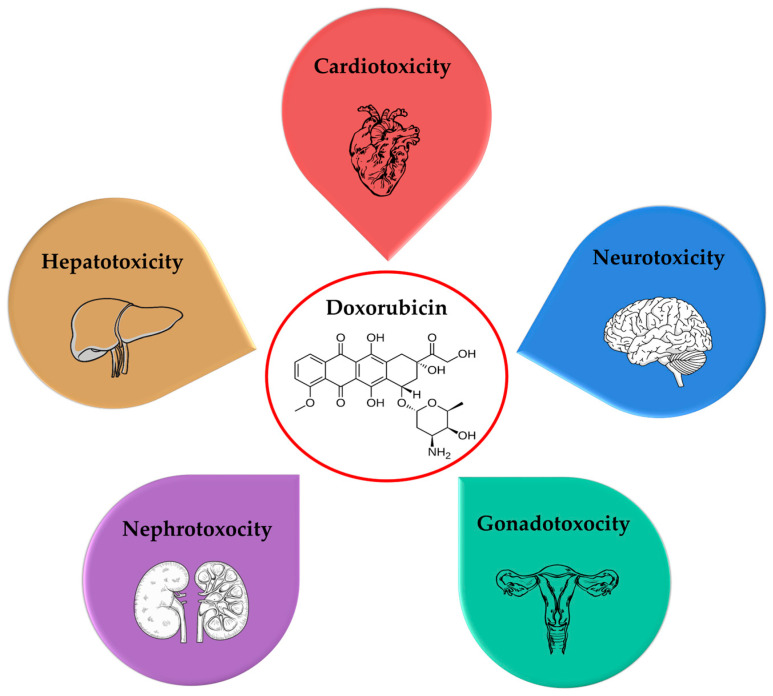
The most common adverse effects of doxorubicin.

The most common adverse effects of doxorubicin are cardiotoxicity, neurotoxicity, hepatotoxicity, nephrotoxicity, and gonadotoxicity (Figure 1). The mechanisms of action of doxorubicin, related to their occurrence with a special focus on oxidative stress, as well as some recent in vivo evidence, are summarized in the next few subsections.

### 3.1. Cardiotoxicity

The wide application of doxorubicin in the treatment of cancer is related to increased survival rate. However, the use of the anthracycline antibiotic results in cardiac dysfunction related to asymptomatic left ventricular dysfunction. The cardiotoxicity of doxorubicin is considered as the most common and severe adverse effect. It is a consequence of processes such as oxidative stress, inflammation, apoptosis, ferroptosis, mitochondrial dysfunction, and calcium dysregulation. Doxorubicin, as a quinone molecule, can be reduced to semiquinone by a variety of enzymes, such as cytosolic xanthine oxidase, nitric oxide synthases, NADH dehydrogenase (complex I), and NADPH (nicotinamide adenine dinucleotide phosphate)-dependent cytochrome P450 reductases. Thereafter, auto-oxidation occurs, and it is returned to its neutral state (redox cycle). This step leads to the generation of O^2−^ and H_2_O_2_. The superoxide anion can further react with NO (nitric oxide) and form ONOO^−^ (peroxynitrite). Interestingly, doxorubicin can promote its redox cycle via increase in the mRNA (messenger ribonucleic acid) expression and enzymatic activity of xanthine oxidase as well as an upregulation of nitric oxide synthase. It is also known that doxorubicin reduces the expression of antioxidant enzymes, which results from the downregulation of Nrf2 (nuclear factor erythroid 2-related factor 2) as well as the inhibition of the mitochondrial sirtuin Sirt3. Moreover, antioxidant enzymes such as glutathione peroxidase and catalase are fundamentally less abundant in cardiomyocytes, making them more sensitive to oxidative stress [4,5,6,7,8].

The long-term cardiotoxic effects of doxorubicin have been evaluated in vivo in male B6C3F_1_ mice by Desai et al. [9]. The animals were treated with 6, 9, 12, and 24 mg/kg total cumulative doxorubicin doses or saline as control, and their cardiac function was assessed via echocardiography 1, 4, 10, 17, and 24 weeks after the treatment. The findings suggested that the mechanism of delayed-onset cardiotoxicity are related to sarcoplasmic reticulum calcium handling and apoptosis. Li et al. revealed that the underlying mechanism of chronic cardiotoxicity of doxorubicin in male C57 mice is ferroptosis [10]. Anemia was registered in the animals that received a cumulative dose of 15 mg/kg doxorubicin, and after increasing the cumulative dose to 36 mg/kg, iron overload was observed. It was also found that doxorubicin treatment led to reduction in GSH (reduced glutathione) synthesis and downregulation of Nrf2 expression. Therefore, the induction of oxidative stress plays an important role in the regulation of ferroptosis. In another study, a cumulative dosage of 25 mg doxorubicin/kg body weight resulted in significant weight loss and severe cardiac dysfunction in mice [11]. It was shown that doxorubicin decreased the serum levels of superoxide dismutase and increased malondialdehyde content, which proves the role of oxidative stress in doxorubicin-induced cardiotoxicity. The underlying mechanisms of cardiotoxicity, namely oxidative stress and inflammation, were evaluated in an in vivo model of elderly CD-1 male mice [12]. The animals received doxorubicin twice a week, for 3 weeks, and a cumulative dose of 9 mg/kg was reached. The short and longer adverse effects were evaluated one week or two months after the last administration of the drug. In the case of one-week post-administration increased NF-κB (nuclear factor kappa-light-chain-enhancer of activated B cells) p65 expression, iNOS (inducible nitric oxide synthase), IL (interleukin)-33, IL-6, and Bax (B-cell lymphoma-2-associated X protein) expression were registered. Moreover, there was a decrease in IL-1β, p62, microtubule-associated protein 1A/1B-light chain 3 (LC3)-I, and p38 MAPK (mitogen-activated protein kinase). The results after two months showed a significant increase in glutathione peroxidase 1 and Bax expression with persistent cardiac damage and fibrosis, and a decrease in carbonylated proteins, Nrf2, NF-κB p65, myeloperoxidase, LC3-I, and LC3-II expression. All these data confirm that the cardiotoxicity of doxorubicin is a severe problem which affects the patient’s life long after the treatment.

### 3.2. Neurotoxicity

Neurotoxicity is the other frequently mentioned side effect of the therapy with doxorubicin. However, the penetration of doxorubicin through the blood–brain barrier (BBB) is limited due to the effect of P-glycoprotein efflux pump, and its neurotoxicity is related mostly to peripheral action. Interestingly, since the production of ROS and pro-inflammatory cytokines in cancer patients is significantly increased, the function of the blood–brain barrier is hindered, leading to accumulation of doxorubicin in the brain. Moreover, the mechanisms are dependent on its dose. In a lower dose, doxorubicin exerts apoptosis via enhancement of the activity of caspase-8, while in higher doses it damages DNA, provokes production of ROS, and depolarizes the neuronal mitochondrial membrane. Oxidative stress plays a pivotal role in the neurotoxicity of doxorubicin. It is known that due to extensive metabolism the neurons are significantly more susceptible to oxidative stress in comparison with other cells. Doxorubicin induces the production of O^2−^ and increases the plasma levels of TNF-α, which is capable of crossing the BBB. This leads to activation of NF-kB inflammatory pathway, increased production of NO in the brain, and mitochondrial dysfunction. It was also found that doxorubicin upregulates autophagy, and damages neuronal lysosomes and progenitor (cells that can divide and differentiate into various types of neurons and glial cells) neuronal degradation pathways [13,14,15,16].

The neurotoxic effects of doxorubicin were recently evaluated in vivo in adult male CD-1 mice. The animals received cumulative doses of 9 or 18 mg/kg. The higher dose disrupted the antioxidant defenses by affecting the glutathione levels and manganese superoxide dismutase expression, and elevated the TNF-α (tumor necrosis factor-α) levels. Interestingly, at this dose the Bax protein, which promotes apoptosis, was decreased and the anti-apoptotic protein Bcl-2 (B-cell lymphoma 2) was increased in hippocampal formation regions. In contrast, when the animals received 9 mg/kg accumulative dose, the levels of Bax protein in both hippocampus and prefrontal cortex as well as p53 (tumor protein p53) expression in CA3 hippocampus region were elevated, pointing at apoptosis as the responsible mechanism [17]. It was also found that the neurotoxic effects of doxorubicin can further worsen its cardiotoxicity. In vivo study on normal adult C57BL/6J male mice showed that doxorubicin decreased the density of cardiac sympathetic innervation in both right and left ventricles, led to thinner and fragmented cardiac sympathetic neurons, reduced the density of neuronal soma in cervical and stellate ganglia, and decreased nerve growth factor content [18]. The effect of doxorubicin on cognitive function was investigated by Alhowail et al. in female albino rats [19]. The animals received an acute dose of the drug, namely 25 mg/kg. Behavior tests such as the Y-maze, novel object recognition test (NORT), and fear-conditioning memory tests showed significantly worsen memory of the animals treated with the anticancer agent. Moreover, doxorubicin treatment led to increase in GluA1 (glutamate receptor ionotropic) subunit protein within the AMPA (amino-3-hydroxyl-5-methyl-4-isoxazole-propionate) receptor and NMDAR (N-methyl-d-aspartate receptors) subunits NR2B, and NR2A expression, causing overactivation of neurons, increased Ca^2+^ influx and concentrations, and apoptosis. The levels of the inflammatory and oxidative stress mediators MDA (malondialdehyde), NF-κB, COX-2 (cyclooxygenase-2), and TNF-α, as well as of the pro-apoptotic proteins Bax and caspase-3 in the brain were also elevated. It was also found that the mechanisms of action of doxorubicin are different depending on the type of treatment, acute or chronic. In another research, the chronic treatment (5 mg/kg weekly) with doxorubicin led to decrease in GluA1 subunit-containing AMPA receptors, leading to impaired synaptic plasticity and spatial memory performance [20]. Another study on healthy female Wistar rats treated with low (2 mg/kg), intermediate (4 mg/kg), and high doses (5 mg/kg) of doxorubicin for 2 or 4 weeks revealed that both the dose and the duration of the treatment play a pivotal role in the behavior and memory of the animals [21]. The authors found that the effects on the anxiety, learning, memory, locomotor function, and exploratory activity are dose- and time-dependent. However, impaired locomotor and exploratory activities were observed in all study groups, meaning that even lower doses affect the movement and behavior of the animals. Moreover, neuroinflammation played a key role in these behavior and cognitive changes.

### 3.3. Hepatotoxicity

The hepatotoxic effect of doxorubicin is mainly related to its pro-oxidant action. As it was mentioned above, it increases the levels of MDA, downregulates Nrf2 gene, and reduces the levels of CAT (catalase), SOD (superoxide dismutase), GST (glutathione S-transferase), NADPH, and HO-1 (hemeoxygenase-1). Moreover, Fe^3+^ can react with doxorubicin, leading to the formation of Fe^2+^ doxorubicin free radical complex. This triggers production of ROS, for instance H_2_O_2_. The production of ROS also leads to lipid peroxidation and impaired lipid metabolism. The elevated levels of oxidative stress also induce inflammation through activation of the NF-kB signaling pathway via inhibition of Sirt1 (Sirtuin 1) protein, which results in increased levels of IL-1B, IL-6, and TNF-α. Furthermore, other mechanisms of hepatotoxicity are related to damage of DNA, mitochondrial dysfunction (decreased ATP/ADP (adenosine triphosphate/adenosine diphosphate) ratio), and apoptosis (upregulation of Bax and p53) [14,22].

The hepatotoxicity of doxorubicin was examined in male Wistar rats that received 2 mg/kg doxorubicin weekly for 6 weeks. Changes in the hepatic metabolism were registered, particularly an increase in 15 metabolites: 6-phosphogluconate, ADP, ATP, betaine, cAMP (cyclic adenosine monophosphate), CDP (cytidine diphosphate), citrate, creatine, GDP (guanosine diphosphate), GSSG (oxidized glutathione), GTP (guanosine triphosphate), ornithine, serine, UDP (uridine diphosphate), and UDP-glucose [23]. There was also an increase in free carnitine, acetyl- and acyl-carnitines, pointing to increased adipose lipolysis and hepatic fatty acid uptake. This confirms the pivotal role of oxidative stress in doxorubicin-induced hepatotoxicity. Sedeman et al. examined the mechanisms of hepatotoxicity of doxorubicin in a diet-induced obesity mouse model with a E0771 triple negative breast cancer-bearing group [24]. This group received three successive dosages of 4 mg/kg doxorubicin which were administered every three days, leading to a cumulative dosage of 12 mg/kg. Significantly increased lipid accumulation was registered in the liver of the obese tumor-bearing mice treated with doxorubicin in comparison with the non-treated with doxorubicin group. Macrovesicular steatosis, sinusoidal dilation, and lobular inflammation were observed in the group of animals which were both obese and received doxorubicin. The mechanisms of hepatotoxicity of doxorubicin were evaluated in vivo in wild-type and FGF1 (fibroblast growth factor 1) knockout mice [25]. The treatment with doxorubicin led to elevation of ALT (alanine aminotransferase) and AST (alanine aminotransferase). Doxorubicin also exerted oxidative stress with a 3-nitrosative modification of multiple proteins and increased MDA content. This is a confirmation of the pivotal role of oxidative stress in the hepatotoxicity of doxorubicin. Moreover, increased cell apoptosis, Bax expression, and caspase 3 cleavage were observed. There was also increased fibrosis due to upregulation of fibrotic marker connective tissue growth factor and α-smooth muscle actin expression. However, these effects were more pronounced in the mice with deletion of FGF1. In both of the study groups, the mRNA and protein expression of pro-inflammatory cytokines ICAM1 (intercellular adhesion molecule 1), VCAM1 (vascular cell adhesion molecule 1), and MCP-1 (monocyte chemoattractant protein-1) were upregulated. These results also showed that endogenous FGF1 deficiency worsens the hepatotoxic effects of doxorubicin. In another study, C57BL/6 J male mice were treated with 20 mg/kg doxorubicin which led to downregulation of Nrf2, NQO1 (NAD(P)H dehydrogenase quinone 1), NMNAT (nicotinamide mononucleotide adenylyl transferase) 1, NMNAT2, and NMNAT3 [26]. As a result, NAD+ (nicotinamide adenine dinucleotide) deficiency was also observed, which resulted in decreased expression of PARP1 (poly-ADP-ribose polymerase1), CD38 (cluster of differentiation 38), and Sirt1. Therefore, the treatment with doxorubicin exerted oxidative stress in the hepatic tissue.

### 3.4. Nephrotoxicity

Among the other toxic reactions of doxorubicin, nephrotoxicity has also been reported. Doxorubicin increases glomerular capillary penetrance and induces tubular degeneration. It is considered that oxidative stress plays the most important role in this toxicity, as well as apoptosis, inflammation, dysregulated autophagic flux, and fibrosis. Doxorubicin infiltrates into mitochondria, causing oxidative stress, DNA damage, and progressive nephron loss. The aforementioned reaction of doxorubicin with NO further provokes apoptosis and inflammation. The last leads to elevated autophagy. Another underlying mechanism is the dysregulation of the renin–angiotensin system. It was reported that doxorubicin increases the generation of angiotensin II, leading to constriction of blood vessels, retention of Na^+^, and water as well as interrupted regulation of glomerular pressure, inflammation, and fibrosis. This also further leads to production of ROS such as H_2_O_2_ [22,27].

The nephrotoxic effects of doxorubicin were evaluated on male and female Sprague–Dawley rats which received intraperitoneal 20 mg/kg doxorubicin with or without previous exercise on treadmill. It was found that the groups treated with doxorubicin showed elevated levels of IP-10 (interferon gamma-inducible protein 10), APG (alpha-1-acid glycoprotein), and NGAL (neutrophil gelatinase-associated lipocalin), indicating inflammation, regardless of exercise. Moreover, only in the male rats treated with doxorubicin, elevated levels of the glycoprotein clusterin, a marker of renal disease, were observed, and the levels of creatinine in urine were normalized in the exercising male group [28]. In another study, healthy male Wistar rats were also treated with doxorubicin intraperitoneally which resulted in increased serum levels of creatinine, urea nitrogen, and β2-microglobulin, as well as increased urine levels of total protein, creatinine, and urea, whereas the serum concentrations of total protein and albumin decreased. Furthermore, the histopathology revealed signs of acute tubular injury while the immunohistochemical studies showed increased levels of the pro-apoptotic protein Bax and decreased levels of anti-apoptotic protein Bcl2, as well as increased expression of COX-2, indicating apoptosis and inflammation, originating from excessive oxidative stress [29].

### 3.5. Gonadotoxicity

Another significant toxic effect of doxorubicin is gonadotoxicity, which is observed in both males and females. The excessive production of reactive oxygen species is pointed out as main mechanism. Other processes related to this toxicity are LPO (lipid peroxidation), inflammation, apoptosis, and autophagy. Regarding the male individuals, elevated levels of 8-hydroxy-20-deoxyguanosine, a marker of oxidative DNA damage, and MDA, as well as reduced levels of antioxidant enzymes such as SOD, CAT, GSH, and GSH-Px (glutathione peroxidase) were observed. Doxorubicin mainly affects the spermatogenesis via disruption of mTOR (mechanistic target of rapamycin) signaling pathway, as well as the sperm motility via reduced ATP levels. Regarding the female individuals, elevated oxidative stress and apoptotic markers (p53, Bax/Bcl2) after doxorubicin treatment are also observed. The estrogenic synthesis is also disrupted via decreased progesterone and pregnenolone levels, which is a result from increased levels of StAR (steroidogenic acute regulatory) protein and decreased levels of cholesterol aromatase (P450arom) [14,30,31,32].

It was found that intraperitoneal administration of doxorubicin (7.5 mg/kg) in sexually matured male Wistar rats led to elevated levels of reactive oxygen and nitrogen species, H_2_O_2_ and LPO, as well as decreased activities of GSH, GST, CAT, SOD, and GPx in testes and epididymis. Moreover, the activities and levels of the inflammation and apoptosis markers NO, MPO (myeloperoxidase), TNF-α, and caspase-3 were significantly elevated in these organs. The levels of intratesticular and serum testosterone as well as the serum follicles stimulating hormone levels were decreased, while the serum prolactin level was increased. The activities of specific testicular marker enzymes ACP (acid phosphatase) and ALP (alkaline phosphatase), as well as G6PD (glucose-6-phosphate dehydrogenase) and LDH (lactate dehydrogenase) were dysregulated. Also, the authors observed a decrease in sperm motility, daily sperm production, and sperm number as well as an increase in sperm abnormalities [33]. The administration of doxorubicin (5 mg/kg intraperitoneal) in adult female Wistar rats resulted in altered IL-1β, TNF-α, and caspase-3 expression as well as levels of MDA. The antioxidant enzymes SOD, CAT, and GSH-Px were also affected [34].

## 4. Novel Approaches for Alleviating the Adverse Reactions of Doxorubicin

**Figure 2:**
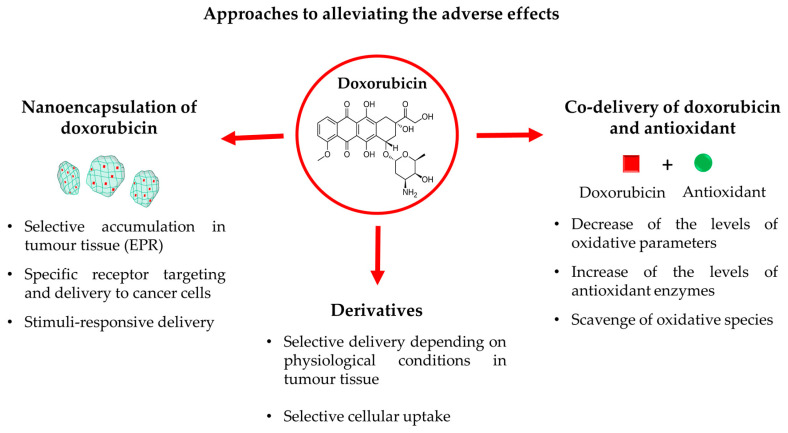
Promising approaches to dealing with the toxicity of doxorubicin: encapsulation of doxorubicin in nanoparticles, co-delivery with antioxidants, and development of derivatives with lower toxicity.

Nowadays, different approaches to dealing with the toxicity of doxorubicin are widely investigated. Some of the most promising strategies are discussed below, including encapsulation of doxorubicin in nanoparticles, co-delivery with antioxidants, and development of its derivatives with lower toxicity (Figure 2). The figure summarizes the mechanisms by which each of these approaches could decrease the toxic effects provoked by doxorubicin-induced oxidative stress. The advantages and limitations of the mentioned approaches are discussed in the following subsections.

### 4.1. Nanoencapsulation of Doxorubicin

The encapsulation of active substances in nanosized drug delivery systems is a well-known innovative approach to increasing pharmacological activities, improving pharmacokinetic properties as well as decreasing toxicity. As shown in Figure 2, the main mechanisms by which nanoencapsulation could reduce toxicity are passive targeting to tumor tissues (1), active receptor targeting and further selective uptake in cancer cells (2), and developing a stimuli-responsive doxorubicin release (3).

The passive targeting is closely related to the phenomenon of enhanced permeability and retention of nanoparticles in tumor tissues (EPR effect). In particular, due to the specific structure of the vascularity of tumors and poor lymphatic drainage, nanoparticles with a diameter less than 200 nm could preferentially cumulate in the tumor site [35]. The passive tumor targeting depends on the physicochemical properties of the nanoparticles, namely their diameter, surface charge, and hydrophilicity. The optimal nanoparticles that could enable passive targeting of doxorubicin solely to tumor tissue should have a prolonged circulation in the bloodstream (e.g., the pegylated surface of nanoparticles helps to avoid phagocytosis) and a diameter smaller than 200 nm to accumulate successfully in the tumor. Thus, the encapsulation of doxorubicin in such nanoparticles could lower the dose that reaches the healthy cells, leading to decreased toxicity. For instance, the toxic effects of PEGylated liposomes loaded with doxorubicin on heart and liver tissue were evaluated in female BALB/c mice bearing 4T1 breast cancer cells [36]. The size of the nanoparticles (104 nm) indicated a potential EPR effect and low toxicity. In the heart tissue of mice treated with the liposomal doxorubicin the levels of the antioxidant enzymes, CAT and SOD were higher compared to the group treated with the free drug. Thus, the results showed a decreased oxidative stress. Histopathological assessment also revealed that the administration of the liposomal drug leads to less side effects on both cardiac and liver tissue than free doxorubicin [36]. Recent studies on human-induced pluripotent stem cell-derived cardiomyocytes, endothelial cells, cardiac fibroblasts, and multi-lineage cardiac spheroids revealed decreased cardiotoxic effects of doxorubicin by its encapsulation in albumin nanoparticles [37]. The authors observed that in the cardiomyocytes the non-loaded doxorubicin showed loss of cell integrity, but not the encapsulated one. The cells treated with the encapsulated drug maintained their viability and metabolism. Cytotoxicity studies were also conducted on cardiomyocytes derived from patient-specific stem cells harboring a single nucleotide polymorphism (rs2229774) in RARG (retinoic acid receptor-γ) and on a corresponding isogenic control cardiomyocyte line. Reduced cardiotoxicity was observed for the loaded doxorubicin in the patient-specific RARG mutant cardiomyocytes, which are more susceptible to doxorubicin-induced cardiotoxicity. The contractility of the cardiomyocytes was also maintained after treatment with the encapsulated doxorubicin compared to the free drug. The cardiac spheroids, composed of cardiomyocytes, endothelial cells, and cardiac fibroblasts, co-cultured in 3D spherical format, showed less susceptibility to the toxic effects of the encapsulated drug compared to free doxorubicin. Maintained contractility at a higher level as well as improved calcium handling were also observed for the encapsulated doxorubicin treatment. At the same time, the anticancer effect of the loaded doxorubicin on human breast cancer epithelial cells BT-549 was retained [37]. Doxorubicin was also loaded into poly(lactic-co-glycolic acid) (PLGA) nanoparticles (252 nm) which resulted in reduced cardiotoxic effects in Wistar rats [38]. Mokhtar et al. developed pectin nanoparticles loaded with doxorubicin. The authors stated that the small size of the pectin particles provides EPR effect and suggested that pectin could be considered as a tumor-targeting agent, since various types of tumors have overexpression of β-galactoside-binding protein which could bind pectin [39]. They observed ameliorated cardiac creatine kinase and LDH, decreased MDA levels, improved GSH-Px, GSH, SOD, TNF-α, and MCP-1, depletion in caspase-3 and p53, and an elevation of Bcl2 level in comparison with the treatment with free doxorubicin. The successful reduction of doxorubicin toxicity through passive targeting is confirmed by the implementation of two products in clinical practice, namely Doxil (pegylated liposomes, diameter 100 nm) and Myocet (non-pegylated liposomes, diameter 180 nm).

Active targeting of doxorubicin-loaded nanoparticles is another mechanism by which nanoencapsulation could ensure its low toxicity to healthy cells. Active targeting is achieved through decoration of the nanoparticle surface with ligands that bind specifically to receptors overexpressed on tumor cells but absent on healthy cells. Thus, the receptor binding would increase the selective doxorubicin delivery to tumor cells and further reduce drug toxicity. For instance, doxorubicin has been loaded in pH-sensitive liposomes coated with folic acid aiming to ensure specific targeting of breast cancer cells [40]. The study reported an increased cellular uptake of the liposomes in the MDA-MB-231 (FR+) breast cancer cell line as well as decreased systemic toxicity in healthy female BALB/c mice even at 20 mg/kg dose in comparison with non-loaded doxorubicin. The biochemical parameters indicative of renal, hepatic, and cardiac toxicity were also evaluated. The levels of urea and creatinine, AST, and CK-MB were increased in the groups treated with free doxorubicin and remained in normal levels in the groups treated with the loaded drug. Only the levels of ALT were increased in both groups. Another research group developed RGD peptide-modified magneto-liposomes loaded with doxorubicin and indocyanine green [41]. The RGD peptide could specifically bind to integrin receptors overexpressed on tumor cells, while the dye indocyanine green was used for diagnostic purposes. It was found that the uptake of the targeted liposomes was higher in human glioblastoma U373MG cells with high integrin αvβ3 receptor expression. The toxicity of the system was tested on healthy BALB/c mice. No change was observed in ALT, AST, and CK-MB in the group treated with the encapsulated doxorubicin compared to the free drug, suggesting a lack of liver and heart toxicity. The antioxidant enzymes CAT and GST were kept at normal levels after treatment with liposomes while a decrease was observed after applying the free doxorubicin. Histopathological analyses confirmed these results. Yang et al. developed nanoparticles based on a prodrug of doxorubicin conjugated to cathepsin B-cleavable peptide *(Phe-Arg-Arg-Gly)* [42]. The prodrug nanoparticles cleaved and delivered doxorubicin only in cathepsin B-overexpressing cancer cells, leading to cancer cell-specific cytotoxicity. The toxicity of the system was tested on colon tumor (CT26)-bearing Balb/c mice. In comparison with the group treated with the non-loaded doxorubicin, the animals administered with the encapsulated drug showed no decrease in their weight, extended lifespan, normal serum levels of alanine aminotransferase, blood urea nitrogen and LDL (low-density lipoptotein), number of reticulocytes, and lymphocytes. Histological analysis of heart, kidney, and liver showed damaged areas only in the group of free doxorubicin. The expression of toll-like receptor 4 mRNA (a mediator of inflammation) in H9C2 cardiomyocytes in vitro was also increased after administration of free doxorubicin with lack of alteration for the encapsulated drug. The loaded doxorubicin also showed significantly reduced proinflammatory cytokines in the healthy mice, namely TNF-α, IL-6, and iIL-1β, compared to the free drug. Another study reported development of doxorubicin-loaded liposomes modified with a lipid stearic acid-peptidomimetic conjugate, aiming to ensure targeted delivery in non-small-cell lung cancer and breast cancer cell lines overexpressing human epidermal growth factor receptor-2 (HER2) [43]. Histology on lungs, liver, heart, kidney, and spleen revealed that the groups treated with the liposomal doxorubicin showed no histological changes as compared to free doxorubicin. Mal et al. loaded doxorubicin in mesoporous silica nanoparticles decorated with folic acid or hyaluronic acid [44]. The toxicity of the system was evaluated in a 4T1 orthotopic tumor model in mice. The animals that received free doxorubicin showed 13-fold reduction in weight as well as a damaged spleen, compared to the control group, while the mice administered with the loaded drug in both the folic and hyaluronic acid-targeted nanoparticles showed neither decrease in their weight nor spleen damage.

Encapsulation in stimuli-responsive nanoparticles is another mechanism that could limit doxorubicin toxicity. In particular, the specific conditions in both the tumor tissue and tumor cells (pH, temperature, oxidative stress, glutathione concentration) could enable preferential delivery of doxorubicin. The latter might result in lower distribution of doxorubicin in healthy tissue and cells. Gomes et al. developed pH-sensitive exosome–liposome hybrid nanocarriers of doxorubicin aiming to achieve a pH-induced release of doxorubicin into the acidic tumor microenvironment [45]. Furthermore, the presence of exosomes extracted from breast cancer cells was considered to improve the interaction with the membrane and cell uptake in tumor cells. It was found that the LD_50_ of the liposomal doxorubicin in healthy female Balb/c mice was between 17.5 mg/kg and 20 mg/kg, whereas of the free doxorubicin it was between 12.5 and 15 mg/kg. According to hematological assessments, WBC (white blood cells count), granulocytes (neutrophils, eosinophils and basophils), and agranulocytes (lymphocytes and monocytes) levels as well as the RBC (red blood cells count), amount of HGB (hemoglobin), and HCT (hematocrit) were increased only in the group treated with the free doxorubicin at a dose of 12.5 mg/kg. The cardiotoxic effect was assessed by measuring the CK-MB (creatine kinase myocardial band) activity. It was found that the increase in the CK-MB level was higher for the group treated with the free doxorubicin at a dose of 12.5 mg/kg in comparison to the encapsulated drug at a dose of 17.5 mg/kg. Thus, the small diameter (105 nm), the pH responsiveness of the systems, and exosome presence led to lower accumulation in the heart. The side effects of doxorubicin have also been mitigated via encapsulation in pH-sensitive chitosan/P(*N*-isopropylacrylamide-co-acrylic acid) nanogel particles (216 nm) [46]. The study group discovered that the mean survival rate in Ehrlich ascites carcinoma-bearing mice was increased. Also, the nanoparticles managed to mitigate oxidative stress (normalized levels of SOD, CAT, and GSH), and inhibit lipid peroxidation (decreased levels of MDA) and NO formation compared to the non-loaded doxorubicin. Moreover, the hepatic DNA deterioration was hindered, the liver (ALT, AST, ALP) and cardiac (CK and CK-MB) enzyme levels were normalized, and the renal complication was ameliorated (decreased levels of serum urea and creatinine). Thus, the pH-responsive delivery provided superior antitumor activity and significant reduction in toxicity at the same time. However, such responsiveness makes the desired delivery weakly effective as it relies on only one endogenous stimulus. In this view, the development of multi-responsive nanosized drug delivery systems appeared as a much more advanced strategy. The development of nanoparticles that possess a redox response is particularly important because the levels of endogenous glutathione are higher in the tumor cells than in healthy cells. By incorporating disulfide bonds the nanoparticles remain stable in the extracellular medium but disintegrate and release the drug in the presence of glutathione via a thiol–disulfide exchange reaction. As mentioned above, the combination of different stimuli is a more efficient strategy for targeted delivery and lower toxicity. Tan et al. developed dual pH/redox-responsive hydroxyethyl starch carrier conjugated with doxorubicin via disulfide and hydrazone bonds [47]. The conjugate self-assembled into nanoparticles (150 nm) in aqueous medium that probably enabled their accumulation in the tumor tissue of H22-tumor-bearing mice. This result correlated with the fact that the nanoparticles did not accumulate in the cardiac tissue (toxic effects were not observed), whereas the treatment with free doxorubicin resulted in pathological changes such as hypertrophy and aggregated inflammatory cardiac muscle cells. Further, the disulfide and hydrazine bonds provided fast intracellular release of doxorubicin in the tumor cells due to their disruption at high glutathione concentration and acidic medium, respectively. Another study group prepared dual pH/redox-responsive polymer-doxorubicin prodrug micelles [48]. The authors observed a reduced in vitro toxicity on normal human embryonic kidney HEK293 cells treated with the double-responsive micelles in comparison with free doxorubicin. Moreover, the toxicity of the nanoparticles was higher in liver cancer cells HepG2 compared to the healthy cells, indicating pH/redox responsive release of doxorubicin. Luo et al. developed pH/redox-responsive micelles based on two polyprodrugs, where doxorubicin was conjugated via pH- and glutathione-sensitive disulfide bonds [49]. The high stability, small size (about 117 nm), and dual responsive properties facilitated high accumulation of the micelles at tumor site and pH/glutathione triggered release. The treatment with free and micellar doxorubicin showed that only 10% of MDA-MB-231-inoculated mice survived with free doxorubicin (24th day) compared to more than 70% with the dual-responsive doxorubicin-loaded micelles.

In conclusion, nanoencapsulation is a strategy that can achieve selective delivery and limit the toxic effects of doxorubicin in healthy tissues. The efficiency of this strategy is enhanced by combining several approaches. In particular, the nanosize and pegylated surface ensure the circulation of nanoparticles in the bloodstream and their accumulation predominantly in the tumor tissue (1), active targeting of receptors that are overexpressed on tumor cells provides successful intracellular transport (2), and stimuli-responsive component triggers the release of doxorubicin under specific conditions (3). Despite the undeniable advantages of nanodelivery, there are still issues that have to be considered. Challenges such as detailed characterization and validation of the nanosystems, as well as some safety concerns, must be addressed.

### 4.2. Co-Delivery of Doxorubicin and Antioxidants

Since the main mechanism of all of the toxic effects of doxorubicin is related to excessive oxidative stress, the simultaneous administration of the anticancer agent and antioxidants could be considered an appropriate strategy to mitigate the aforementioned side effects. The antioxidants could be co-delivered alongside doxorubicin or they could be simultaneously encapsulated with doxorubicin in one drug delivery system.

**Figure 3:**
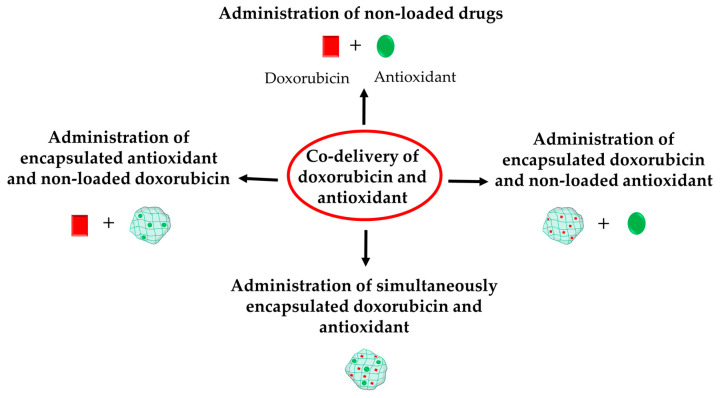
Different possibilities for co-delivery of doxorubicin with antioxidants.

The co-administration of doxorubicin and an antioxidant could be implemented as follows: (1) in their free forms, (2) free form of doxorubicin and nanoencapsulated antioxidant, (3) nanoencapsulated doxorubicin and free antioxidant, or (4) simultaneous encapsulation of doxorubicin and antioxidant in one drug delivery system (Figure 3). The most recent achievements in this section will be categorized depending on the type of administration of doxorubicin and antioxidants.

#### 4.2.1. Administration of Non-Loaded Forms of Doxorubicin and Antioxidant

This strategy is based on the separate preparation and administration of dosage forms containing doxorubicin or certain antioxidants. The main advantage of this strategy is the possibility of adjusting the desired concentrations. The main limitation is the solubility of the selected antioxidant, especially considering that many natural antioxidants are insoluble in water. The non-soluble antioxidant molecules require the use of an appropriate solvent, which provides their dissolved form. Thus, the strategy is easier to implement and more effective if the antioxidant is water soluble. For instance, crocin is a water-soluble carotenoid pigment extracted from Crocus sativus. Abdulkareem Aljumaily et al. tested the effect of crocin on doxorubicin-induced cardiotoxicity in rats by intraperitoneal administration of solutions of doxorubicin and crocin [50]. The administration of doxorubicin alone led to a significant increase in the lipid indices, oxidative stress parameters, and cardiac markers (CK-MB and cardiac troponin I). In contrast, the co-administration of crocin and doxorubicin considerably ameliorated the mentioned parameters in comparison with the doxorubicin group. Histopathological analysis revealed a significant increase in the mean histopathological damage score in the group treated with doxorubicin alone, while the co-administration of crocin with doxorubicin reduced this score in myocardium. The protective effect of other antioxidants against doxorubicin-induced cardiotoxicity was also observed, e.g., mokko lactone [51], ergothioneine [52], quercetin [53], minocycline [54], hersperidin [55], curcumin [56], sinapic acid [57], reduced glutathione [58], dihydroartemisinin [59], and ethoxyquin [60] (Table 1).

Various antioxidants were examined as hepatoprotective agents against doxorubicin-induced liver toxicity, e.g., crocin [61], ginkgetin [62], tannic acid [63], curcumin [56], esculetin [64,65], and luteolin [66]. The hepatoprotective effect of rutin and quercetin (50 mg/kg), alone or in a combination, co-administered with doxorubicin (2 mg/kg) in free form was evaluated in male Wistar rats after administration for five weeks [67]. The serum levels of ALT, AST, ALP, bilirubin, and albumin were significantly increased in the group treated only with doxorubicin. All of the other three groups, which were co-administered with rutin, quercetin, or combination of both, showed normalized levels of all serum parameters. Quercetin showed the best activity in reducing the high ALT and ALP levels and increasing the lowered albumin level. Rutin was most potent in reducing the high AST and bilirubin level. Regarding oxidative stress, the doxorubicin group showed downregulated levels of GSH and increased lipid peroxidation. Only the co-administration with rutin managed to upregulate the GSH levels. The three groups showed a decrease in the lipid peroxidation, with quercetin being the most effective. Moreover, the activities of GPx and GST were significantly decreased in the doxorubicin group. Regarding GPx, the rutin administration increased its activity, while regarding GST, the combination of rutin and quercetin was the most effective. According to conducted histopathological analyses, the co-administration of quercetin and the combination of quercetin and rutin showed improvement in the liver histological features. Namely, histopathological changes were absent and slight vacuolization of hepatocytes was observed. The effect of the combination of the two antioxidants was also the most potent regarding lesion scores related to inflammation, necrosis, activated apoptosis, vascularization of hepatocytes, clear cells of hepatocytes, and karyomegaly of hepatocytic nuclei. The expression of p53 protein (tumor suppressor) and TNF-α (an inflammation marker), which were significantly increased in the doxorubicin group, were normalized in the co-treated groups. Rutin showed the highest activity against expression of p53, while the combination of the two antioxidants was more effective against TNF-α. The expression of Nrf2 (a transcription factor responsible for antioxidant defense) was negative in the doxorubicin group while rutin and quercetin, especially rutin, increased its expression.

The protective effect of *Chlorella vulgaris* against doxorubicin-induced male reproductive toxicity was tested in Wistar rats. The animals received normal saline (control), doxorubicin (3 mg/kg/weekly intraperitoneally), *Chlorella vulgaris* powder (300 mg/kg/day orally), or both doxorubicin and *Chlorella vulgaris* [68]. The doxorubicin group showed reduced water and food intake, leading to significant weight loss. Moreover, a significant decrease in testes weight, length, and diameter was also observed. Histopathological studies revealed that in the doxorubicin group there were abnormalities in the seminiferous epithelium and germ cells, as well as basement membrane thickening and interstitial hyperemia. The authors also observed decreased spermatozoa, disturbed spermatogenesis, absence of primary and secondary spermatocytes, sperm maturation arrest, and disturbance in the typical morphology of the testes. In the group co-treated with the antioxidant, an improved testes structure and increased spermatogenesis were registered. In addition, in the co-treated group there was a significant increase in the number of spermatogonia, primary spermatocytes, early and late spermatid cells, the height of seminiferous epithelium, and the diameter of seminiferous tubules. Similar results were observed regarding the sperm parameters mean sperm count, percentage of motility, viability, and normal sperm morphology. The decreased by doxorubicin serum testosterone levels were also relatively normalized by the co-administration. The levels of Bax and p53 (markers of apoptosis) were significantly altered in the doxorubicin group, while the simultaneous application of *Chlorella vulgaris* led to a significant decrease. The levels of the oxidative stress markers, total antioxidant capacity, and oxidative stress index were also normalized after co-administration. Other recent examples of antioxidants with protective potential against doxorubicin-induced reproductive toxicity are quercetin [69] (male and female) and vitamin E (female) [70], gallic acid (female) [71], propolis (male) [72], resveratrol (female) [73], isorhamnetin (male) [74], and diosmin (male) [75].

Gad et al. tested the potential of co-administration of naringin along with doxorubicin for alleviating doxorubicin-induced nephrotoxicity in adult male albino Wistar rats [76]. The antioxidant was administered orally (100 mg/kg/daily) 10 days before doxorubicin treatment. Doxorubicin administration resulted in increased levels of creatinine, urea, and uric acid. Furthermore, doxorubicin led to a significant decrease in nephrin (that regulates the kidney function and maintains the filtration barrier) and podocin (it participates in maintaining the filtration barrier, too) in renal tissue. The administration of naringin normalized the redox balance via amelioration of ROS levels, oxidative stress markers (MDA, protein carbonyl and 8-hydroxydeoxyguanosine), and antioxidants (GSH, GSH-Px and GR (glutathione reductase)). The increase of the inflammatory mediators IL-6, IL-1β, TNF-α, and NF-κB was also prevented by naringin and the anti-inflammatory IL-10 was upregulated. The alleviation of oxidative stress resulted also in decreased expression of TGF-β1 (transforming growth factor β1) and the apoptotic protein caspase-3. Other antioxidants, with protective potential against doxorubicin-induced nephrotoxicity are honey, royal jelly and propolis (combination) [77], Ceratonia siliqua pods (Carob) methanol extract [78], hesperetin [79], geraniol [80], resveratrol [81], myricetin [82], and gossypetin [83].

The potential of coenzyme Q10 to alleviate doxorubicin-induced neurotoxicity was investigated in female rats [84]. The animals received 200 mg/kg coenzyme Q10 for 21 days and 4 mg/kg doxorubicin on day 7 and day 14. The administration of doxorubicin alone led to alterations in locomotor activity and anxiety levels, an increase in acetylcholinesterase levels, and alterations in the levels of MDA, protein carbonyl, glutathione peroxidase, and total antioxidant capacity. The co-delivery of coenzyme Q10 resulted in normalizing acetylcholinesterase activity, leading to alleviated behavioral alterations. The neuroprotective effect of alpha-lipoic acid was also examined in Sprague–Dawley rats [85]. The animals were administered with 2 mg/kg/weekly doxorubicin and 50, 100, or 200 mg/kg alpha-lipoic acid for 4 weeks. The co-administration of alpha-lipoic acid with doxorubicin resulted in alleviated memory impairment and normalized the levels of hippocampal antioxidants. In addition, the co-administration reduced the oxidative and inflammatory processes via upregulation of the levels of Nrf2 and HO-1. The protective potential of other antioxidants against doxorubicin-induced neurotoxicity, namely galantamine [86,87], curcumin [88], quercetin [89,90], juglanin [91], melatonin [92], propolis [93], diphenyl diselenide [94], and luteolin [66] was also reported.

**Table 1:** Recent examples of mitigating doxorubicin toxicity via co-delivery of doxorubicin and an antioxidant.

Type of Toxicity	Antioxidant	Reference
Cardiotoxicity	Crocin	[50]
	Mokko lactone	[51]
	Ergothioneine	[52]
	Quercetin	[53]
	Minocycline	[54]
	Hesperidin	[55]
	Curcumin	[56]
	Sinapic acid	[57]
	Reduced glutathione	[58]
	Ethoxyquin	[60]
	Dihydroartemisinin	[59]
Hepatotoxicity	Rutin and quercetin	[67]
	Crocin	[61]
	Ginkgetin	[62]
	Tannic acid	[63]
	Curcumin	[56]
	Esculetin	[64,65]
	Luteolin	[66]
Gonadotoxicity	*Chlorella vulgaris*	[68]
	Quercetin	[69,70]
	Vitamin E	[70]
	Gallic acid	[71]
	Propolis	[72]
	Resveratrol	[73]
	Isorhamnetin	[74]
	Diosmin	[75]
Nephrotoxicity	Naringin	[76]
	Honey, royal jelly and propolis	[77]
	Ceratonia siliqua extract	[78]
	Hesperetin	[79]
	Geraniol	[80]
	Resveratrol	[81]
	Myricetin	[82]
	Gossypetin	[83]
Neurotoxicity	Coenzyme Q10	[84]
	Alpha-lipoic acid	[85]
	Galantamine	[86,87]
	Curcumin	[88]
	Quercetin	[89,90]
	Juglanin	[91]
	Melatonin	[92]
	Propolis	[93]
	Diphenyl diselenide	[94]
	Luteolin	[66]

#### 4.2.2. Administration of Free Doxorubicin and Encapsulated Antioxidant

There are several studies that examined simultaneous administration of free doxorubicin and an antioxidant encapsulated in nanoparticles. Since most of the attractive antioxidants are poorly soluble in water, the idea of this strategy is to improve solubility via encapsulation in nanoparticles. For instance, quercetin is a poorly soluble flavonoid which hinders its effective absorption. Thus, Soliman et al. developed chitosan–quercetin nanoparticles, which were co-administered with non-encapsulated doxorubicin in rats aiming to reduce its cardiotoxicity [95]. The quercetin nanoparticles normalized the increased by doxorubicin levels of NO, MDA, IL-1β, TNF-α, caspase-3, total cholesterol, triglycerides, VLDL-c (very-low-density lipoprotein-cholesterol), LDL-c (low-density lipoprotein-cholesterol), CK-MB, AST, LDH, troponin I, and annexin-V. The co-administration of the encapsulated quercetin also normalized the decreased levels of GSH, GST, GSH-Px, and SOD. The authors concluded that quercetin was responsible for the scavenging of superoxide radicals and reduced myocardial damage. Quercetin and Zn^2+^, incorporated in bovine serum albumin nanoparticles, also showed cardioprotective effect when administered with doxorubicin [96]. Xanthohumol, a chalconoid extracted from Humulus lupulus, was encapsulated in poly(lactic-co-glycolic acid) nanoparticles, coated with an erythrocyte membrane, and co-delivered with free doxorubicin in vivo [97]. The authors observed a significant reduction of ROS-dependent ferroptosis and an improvement of the cardiac dysfunction. It was concluded that xanthohumol reduced the doxorubicin-induced cardiotoxicity by inhibiting ferroptosis. Other study groups reported alleviated doxorubicin-induced cardiotoxicity by co-administration of doxorubicin with Moringa oleifera leaf extract loaded in niosomes [98], icariin nanoemulsion [99], and berberine encapsulated in micelles [100] or solid lipid nanoparticles [101] (Table 2).

Aiming to reduce the hepatotoxicity of doxorubicin, Mohamed et al. developed zein nanoparticles co-loaded with cinammonaldehyde and naringin [102]. The protective potential of the nanosystem was tested on female Swiss albino mice. The treatment with the encapsulated antioxidants led to a significant increase in the levels of GSH and catalase (CAT) as well as a significant decrease in the MDA, MPO, and TGF-β1 levels compared to the doxorubicin group. Moreover, the body weight of the mice was maintained, while doxorubicin alone led to a significant decrease. Regarding liver and kidney function, the loaded antioxidants normalized the serum levels of ALT, AST, and creatinine which were decreased by doxorubicin. The co-administration of thymoquinone loaded in chitosan nanoparticles along with doxorubicin in male albino rats also resulted in hepatoprotection [103]. Turmeric extract loaded in selenium nanoparticles alleviated doxorubicin hepatotoxicity in mice, too [104]. Decreased levels of MDA, NO, IL-1β, TNF-α, and NF-κB p65; elevated levels of SOD, CAT, GPx, GR, GSH and mRNA expression levels of Nrf-2; and downregulation of iNOS gene expression were observed. These results were confirmed by histological examination.

Fu et al. encapsulated eugenol in methoxy-poly(ethylene glycol)-poly(lactide-co-glycolide) nanoparticles (mPEG-PLGA-NPs) and evaluated the potential of the system to alleviate doxorubicin-induced male reproductive toxicity in vitro and in vivo [105]. In spermatogonia cell line GC-1, the system significantly reduced doxorubicin-induced production of ROS and inflammatory factors, and regulated the expression of the mitochondrial autophagy protein PINK1 and meiosis-related protein SCP3. In Kunming mice, the encapsulated eugenol significantly increased sperm motility, and reduced apoptosis and oxidative stress in the testes. Pleurotus eryngii extract was loaded in chitosan nanoparticles and its protective potential against doxorubicin-induced testicular damage was evaluated in male rats [106]. The co-administration resulted in alleviated testicular damage via reduced total oxidative status and normalized expression of caspase 3 and DRP1-MFN2 (dynamin-related protein 1). Curcumin was also loaded in selenium nanoparticles and the co-administration with doxorubicin led to protective effects against doxorubicin toxic effects in healthy adult female rats as a result of antioxidant, anti-inflammatory, and anticancer activities [107].

Regarding the nephrotoxicity of doxorubicin, the protective potential of resveratrol loaded in liposomes was tested in male Wister rats [108]. The authors observed elevation in serum urea, creatinine, renal lipid peroxide levels, endoglin expression, KIM-1 (kidney injury molecule-1), and beclin-1 in the doxorubicin group. The renal podocin, mTOR expression, and GSH levels were decreased, and the DNA fragmentation was markedly increased. The treatment with resveratrol managed to ameliorate these parameters. In another study hesperidin was loaded into chitosan nanoparticles and the nephroprotective potential of the system was tested on male albino mice [109]. The pretreatment with the system resulted in lowered kidney enzyme and MDA content, and increased antioxidant enzyme activities (CAT and SOD). Moreover, suppression of TNF-α, IL-1β, VEGF (vascular endothelial growth factor), NF-κB, caspase-3, Bax, and TGF-β1 levels were observed. The administration of the loaded hesperidin increased the gene expression of Sirt1, Bcl-2, VEGF, HIF1-α (hypoxia-inducible factor 1 alpha), and KIM-1.

The antioxidant piperine was loaded in zeolitic imidazolate nanoparticles and its protective potential against doxorubicin-induced neurotoxicity was tested in a zebrafish model [110]. The nanoparticles managed to reverse the impaired cognitive activity, the downregulation of spatial memory and locomotor activity, the downregulation in GSH and SOD levels as well as the increased LPO, AChE (acetylcholinesterase), and TNF-α levels, all of which were observed for the doxorubicin group.

**Table 2:** Recent examples of mitigating doxorubicin toxicity via co-delivery of free doxorubicin and encapsulated in nanoparticles antioxidant.

Type of Toxicity	Antioxidant	Nanoparticles	Reference
Cardiotoxicity	Quercetin	Chitosan nanoparticles	[95]
	Quercetin and Zn^2+^	Bovine serum albumin nanoparticles	[96]
	Xanthohumol	Poly(lactic-co-glycolic acid) (PLGA) nanoparticles, coated with an erythrocyte membrane	[97]
	Moringa oleifera leaf extract	Niosomes	[98]
	Icariin	Nanoemulsion	[99]
	Berberine	Vitamin E-TPGS micelles Solid lipid nanoparticles	[100,101]
Hepatotoxicity	Cinammonaldehyde and naringin	Zein nanoparticles double coated with sodium caseinate and lactoferrin	[102]
	Thymoquinone	Chitosan nanoparticles	[103]
	Turmeric extract	Selenium nanoparticles	[104]
Gonadotoxicity	Eugenol	Methoxy-poly(ethylene glycol)-poly(lactide-co-glycolide) nanoparticles	[105]
	Pleurotus eryngii extract	Chitosan nanoparticles	[106]
	Curcumin	Selenium nanoparticles	[107]
Nephrotoxicity	Resveratrol	Liposomes	[108]
	Hesperidin	Chitosan nanoparticles	[109]
Neurotoxicity	Piperine	Zeolitic imidazolate metal organic frameworks	[110]

#### 4.2.3. Administration of Encapsulated Doxorubicin and Free Antioxidant

The other possibility is the co-administration of encapsulated in nanoparticles doxorubicin with free antioxidant. The idea behind the encapsulation of the anticancer agent is to ensure its selectivity to tumor cells and to decrease its toxicity. In addition, encapsulation of doxorubicin could improve its stability upon light exposure. However, this strategy is not often applied since most antioxidants possess hydrophobic properties, resulting in poor biopharmaceutical properties. Therefore, the encapsulation of the antioxidants is a more preferred strategy.

Dorostkar et al. loaded doxorubicin in liposomes and tested the protective potential of simultaneous administration with free quercetin in vivo [111]. The hydrophobic quercetin was administered by gavage whereas doxorubicin was administered intraperitoneally. The co-administration of liposomal doxorubicin with free quercetin to male Wistar rats resulted in decreased weight loss and levels of CK-MB, LDH, and MDA. Moreover, increased activity of the antioxidant enzymes GSH-Px, CAT, and SOD in the left ventricle was observed. The expression of NOX1 (NADPH oxidase 1), Rac1 (Rac family small GTPase 1 protein), Rac1-GTP (active Rac family small GTPase 1 protein, bound to guanosine triphosphate), Sirt3, and Bcl-2 proteins was also normalized. Another study group developed chitosan nanoparticles loaded with doxorubicin and co-administered them with propionic acid in rats [112]. The co-administration resulted in normalized levels of GST, MDA, LDL-c, and the expression PPARγ (peroxisome proliferator-activated receptor γ) in heart tissues, as well as lack of alteration of P waves in electrocardiography.

#### 4.2.4. Double Encapsulation of Doxorubicin and Antioxidants in Nanoparticles

The simultaneous encapsulation of doxorubicin and antioxidant in one drug delivery system is the most effective approach for co-administration. This approach combines the advantages of specific delivery of the loaded doxorubicin with a higher solubility of the co-loaded antioxidant. Thus, this approach marks a significant improvement in the biopharmaceutical characteristics of both the anticancer agent and the antioxidant. In addition, this strategy significantly facilitates the administration.

Resveratrol is a natural polyphenol, exerting significant antioxidant effects, with quite poor aqueous solubility. In this view, its encapsulation in nanoparticles could improve its solubility and antioxidant potential. Reduced in vitro, cardiotoxicity in H9c2 cardioblasts was registered for double encapsulation of doxorubicin with resveratrol in mixed Pluronic P123 and F127 micelles [113]. The micelles (26 nm) contributed to the higher protection of cardioblasts due to the ability of enhancing cell uptake as well as inhibiting P-gp efflux. In addition, the developed micelles improved the solubility of resveratrol, which probably enabled the intracellular transport and protective action in the cells. Chitosan–albumin nanogel particles were also double-loaded with resveratrol and doxorubicin (mean diameter of 30 nm) [114]. In order to be loaded in the chitosan–albumin nanoparticles, resveratrol was initially included in a complex with hydroxypropyl-β-cyclodextrin. The encapsulation of resveratrol into the nanogel provided cardio- and neuroprotective effects in H9c2 cardioblasts and SH-SY5Y neuroblastoma cells, respectively. For comparison, the treatment of both types of cells with non-encapsulated resveratrol did not exert such protective effect. Pogorzelska et al. formulated liposomes with doxorubicin and sulforaphane [115]. In vivo in A 4T1-implanted murine breast tumor female Balb/c mice, the double drug delivery resulted in cardioprotective, nephro- and hepatoprotective effects. The administration of the double loaded system led to a decrease in weight loss and normalization of the levels of HGB, RBC, HCT, AST, and urea. Epigallocatechin, a well-known polyphenol with strong antioxidant activity, was evaluated as a co-loading agent for reduction of doxorubicin cardiotoxicity [116,117] (Table 3). For example, polyethyleneimine nanoparticles co-loaded with doxorubicin and epigallocatechin reduced in vitro cardiotoxicity in H9c2 cardioblasts and normalized levels of CK-MB, LDH, SOD, and MDA in male nude mice [116].

**Table 3:** Recent examples of mitigating doxorubicin toxicity via double encapsulation of doxorubicin and antioxidant.

Type of Toxicity	Antioxidant	Type of Double-Loaded Nanoparticles	Reference
	Resveratrol	Pluronic P123-F127 micelles	[113]
Cardiotoxicity	Sulforaphane	Liposomes	[115]
	Epigallocatechin	Folic-acid coated polyethyleneimine (PEI) nanoparticles	[116]
	Epigallocatechin-3-gallocarboxylate	L-cysteine-epigallocatechin-3-gallocarboxylate-nanoparticles	[117]
Hepatotoxicity	Sulforaphane	Liposomes	[115]
	Zinc	Zinc oxide nanoparticles	[118]
Nephrotoxicity	Sulforaphane	Liposomes	[115]
	Salvianolic acid A	Nanostructured lipid carriers	[119]
	Zinc	Zinc oxide nanoparticles	[118]
Neurotoxicity	Resveratrol	Chitosan–albumin nanoparticles	[114]

### 4.3. Derivatives

The use of derivatives of doxorubicin that possess lower toxicity is another strategy for improvement of the therapy. For instance, (6-maleimidocaproyl) hydrazone derivative of doxorubicin was developed and further loaded in a byopolimeric carrier consisting of a cell-penetrating peptide (SynB1) (which promotes tumor and cellular uptake) and thermally responsive elastin-like polypeptide (ELP) [120]. The administration in MDA-MB-231 tumor-bearing mice showed a 6-fold increase in tumor/heart ratio compared to free doxorubicin, meaning preferential accumulation in tumors. Further, the pH-sensitive hydrazone linker provides doxorubicin delivery in the acidic tumor environment which is a prerequisite for reduced toxicity on other tissues. Another study developed hybrid cyclic-linear peptide [R5K]W7A and linear peptide R5KW7A derivatives of doxorubicin through a glutarate linker [121]. The [R5K]W7A derivative showed minimal cytotoxicity to normal kidney (LLC-PK1, 5–7%) and heart cells (H9c2, <9%) at concentrations of 5–10 μM. In comparison, free doxorubicin at 5 μM concentration decreased the viability of kidney and heart cells by 85% and 44%, respectively. The authors pointed out that the developed hybrid cyclic-linear peptide conjugate possessed minimal uptake (lower than free doxorubicin) in the healthy kidney and heart cells that minimized doxorubicin toxicity. Lages et al. conjugated doxorubicin to α-tocopherol succinate via amide or hydrazone bond which was further incorporated in a nanostructured lipid carrier [122]. The conjugate formed via hydrazone bond showed a pH-sensitive release of doxorubicin enabled at acidic conditions. Further in vivo studies revealed that this conjugate attenuated the doxorubicin-induced short-term cardiotoxic effects, e.g., impaired left ventricular systolic function. Reduced heart and liver toxicity was also observed. The issue with this strategy, however, is that the derivatization of doxorubicin requires a series of experiments, including theoretical (to select the most appropriate derivative to be synthesized), toxicological (since a new molecule is synthesized), and pharmacological (as the resulting derivative may exhibit an altered antitumor effect compared to pure doxorubicin) experiments.

## 5. Closing Remarks and Perspectives

Despite the progress in the development of multifunctional nanoparticles as carriers of doxorubicin, the incidence of side effects is still a challenge. One of the reasons could be the change of characteristics of the nanoparticles under in vivo conditions. For instance, detachment of the targeting ligands from the surface of the decorated nanoparticles would limit selective delivery of doxorubicin in the target cells. Disaggregation of nanoparticle structure, e.g., micelles upon dilution, would release the drug in the bloodstream, enabling it to reach all tissues. In this regard, a possible solution could be the development of carrier-free nanoparticles combining doxorubicin and the protective agent. The co-assembly of doxorubicin and an antioxidant would bring the advantages of nanoparticles (e.g., EPR effect) but would eliminate the need for a carrier. The differences in endogenous conditions in different patients is another reason for the failure of existing strategies to reduce toxicity. In this regard, the development of nanoparticles coated with homotypic membrane from specific cancer cells is a new strategy for selective delivery to the target cells. This method would provide highly reliable targeting and limitation of toxic effects in other tissues. Thus, novel approaches to mitigating the toxic effects of doxorubicin should be developed as reduced toxicity is crucial for the success of anticancer therapies.

## Data Availability

All data are contained within the article.

## Abbreviations

The following abbreviations are used in this manuscript:

AChE	acetylcholinesterase
ACP	acid phosphatase
ADP	adenosine diphosphate
ALP	alkaline phosphatase
ALT	alanine aminotransferase
AMPA	amino-3-hydroxyl-5-methyl-4-isoxazole-propionate receptor
APG	alpha-1-acid glycoprotein
AST	aspartate aminotransferase
ATP	adenosine triphosphate
Bax	B-cell lymphoma-2-associated X protein
BBB	blood-brain barrier
Bcl-2	B-cell lymphoma 2
cAMP	cyclic adenosine monophosphate
CAT	catalase
CD 38	cluster of differentiation 38
CDP	cytidine diphosphate
CK	creatine kinase
CK-MB	creatine kinase myocardial band
COX-2	cyclooxygenase-2
DNA	deoxyribonucleic acid
DRP1-MFN2	dynamin-related protein 1
EPR	enhanced permeability and retention
FGF1	fibroblast growth factor 1
G6PD	glucose-6-phosphate dehydrogenase
GDP	guanosine diphosphate
GluA1	glutamate receptor ionotropic, AMPA
GR	glutathione reductase
GSH	reduced glutathione
GSH-Px	glutathione peroxidase
GSSG	oxidised glutathione
GST	glutathione S-transferase
GTP	guanosine triphosphate
H_2_O_2_	hydrogen peroxide
HCT	hematocrit
HGB	hemoglobin
HIF1-α	hypoxia-inducible factor 1 alpha
HO-1	hemeoxygenase-1
ICAM1	intercellular adhesion molecule 1
IL	interleukin
iNOS	inducible nitric oxide synthase
IP-10	interferon gamma-inducible protein 10
KIM-1	kidney injury molecule-1
(LC3)-I	microtubule-associated protein 1A/1B-light chain 3
LDH	lactate dehydrogenase
LDL	low-density lipoptotein
LDL-c	low-density lipoprotein-cholesterol
LPO	lipid peroxidation
MAPK	mitogen-activated protein kinase
MCP-1	monocyte chemoattractant protein-1
MDA	malondialdehyde
MPO	myeloperoxidase
mRNA	messenger ribonucleic acid
mTOR	mechanistic target of rapamycin
NAD+	nicotinamide adenine dinucleotide
NADPH	nicotinamide adenine dinucleotide phosphate
NF-κB	Nuclear factor kappa-light-chain-enhancer of activated B cells
NGAL	neutrophil gelatinase-associated lipocalin
NMDAR	*N*-methyl-d-aspartate receptors
NMNAT	nicotinamide mononucleotide adenylyl transferase
NO	nitric oxide
NOX1	reduced nicotinamide adenine dinucleotide phosphate (NADPH) oxidase 1
NQO1	reduced nicotinamide adenine dinucleotide phosphate (NAD(P)H) dehydrogenase quinone 1
Nrf2	Nuclear factor erythroid 2-related factor 2
O^2−^	superoxide anion
•OH	hydroxyl radicals
ONOO^−^	peroxynitrite
p53	tumor protein p53
PARP1	poly-ADP (adenosine diphosphate)-ribose polymerase1
PPARγ	peroxisome proliferator-activated receptor γ
RARG	retinoic acid receptor-γ
RBC	red blood cells count
RNA	ribonucleic acid
ROS	reactive oxygen species
Sirt1	Sirtuin 1
Sirt3	Sirtuin 3
SOD	superoxide dismutase
StAR	steroidogenic acute regulatory protein
TGF-β1	transforming growth factor β1
TNF-α	tumor necrosis factor-α
UDP	uridine diphosphate
VCAM1	vascular cell adhesion molecule 1
VEGF	vascular endothelial growth factor
VLDL-c	very-low-density lipoprotein-cholesterol
WBC	white blood cells count
